# Ontological approach to the knowledge systematization of a toxic process and toxic course representation framework for early drug risk management

**DOI:** 10.1038/s41598-020-71370-7

**Published:** 2020-09-03

**Authors:** Yuki Yamagata, Hiroshi Yamada

**Affiliations:** 1 Toxicogenomics Informatics Project, National Institute of Biomedical Innovation, Health and Nutrition, 7-6-8 Saito-Asagi, Ibaraki, Osaka 567-0085 Japan; 2 Laboratory for Developmental Dynamics, RIKEN Center for Biosystems Dynamics Research, 2-2-3 Minatojima-minamimachi, Chuo-ku, Kobe, 650-0047 Japan

**Keywords:** Cell biology, Computational biology and bioinformatics, Drug discovery, Genetics, Risk factors

## Abstract

Various types of drug toxicity can halt the development of a drug. Because drugs are xenobiotics, they inherently have the potential to cause injury. Clarifying the mechanisms of toxicity to evaluate and manage drug safety during drug development is extremely important. However, toxicity mechanisms, especially hepatotoxic mechanisms, are very complex. The significant exposure of liver cells to drugs can cause dysfunction, cell injury, and organ failure in the liver. To clarify potential risks in drug safety management, it is necessary to systematize knowledge from a consistent viewpoint. In this study, we adopt an ontological approach. Ontology provides a controlled vocabulary for sharing and reusing of various data with a computer-friendly manner. We focus on toxic processes, especially hepatotoxic processes, and construct the toxic process ontology (TXPO). The TXPO systematizes knowledge concerning hepatotoxic courses with consistency and no ambiguity. In our application study, we developed a toxic process interpretable knowledge system (TOXPILOT) to bridge the gaps between basic science and medicine for drug safety management. Using semantic web technology, TOXPILOT supports the interpretation of toxicity mechanisms and provides visualizations of toxic courses with useful information based on ontology. Our system will contribute to various applications for drug safety evaluation and management.

## Introduction

Liver toxicity is a major cause of attrition in drug development and the withdrawal of drug products. Drugs inherently have the potential to cause injury because they are xenobiotics. However, it is difficult to clarify the mechanisms of toxicity because of their complexity. The liver is the main organ where chemicals are metabolized and eventually excreted. Therefore, significant exposure of liver cells to drug and drug candidates can cause liver dysfunction, cell injury, and liver organ failure^1^. For drug risk management, it is essential to systematize the necessary knowledge from a consistent viewpoint.

In this study, we adopt an ontological approach. Originally, ontology was a branch of philosophy, and the term means ‘existence’^2^. Recently, information science has focused on ontology, because ontology provides a controlled vocabulary for sharing and reusing various data in a computer-friendly manner^3^. There are many biomedical ontologies in ontology repository sites such as the National Center for Biomedical Ontology (NCBO) BioPortal (https://bioportal.bioontology.org). However, none of these ontologies organize toxicity mechanisms. To systematize knowledge concerning toxic mechanisms without ambiguity, we constructed a toxic process ontology (TXPO) focusing on hepatotoxic processes.

To analyse toxicity mechanisms for drug safety management, a unified viewpoint is needed to cope with the range of granularities in the human body. Toxicants can disrupt unexpected functions at the molecular, cellular, and/or tissue levels^1^. There are many biomedical pathway databases, including the Kyoto Encyclopedia of Genes and Genomes (KEGG)^4^, WikiPathways^5^, and Reactome^6^. Because these databases deal with many pathways, one might conclude that they also explain toxicity mechanisms. However, most of them are based on molecule–molecule interactions. Such molecular-centred approaches do not cover cell- or organ-level granularity. In this study, we focus on functioning processes and introduce a functional decomposition approach. We capture a biological system and its parts as sub-systems to describe biological defence functions from a consistent viewpoint across granularities.

Screening in drug discovery requires strategies to find and reduce drug risk before a toxic manifestation. An adverse outcome pathway (AOP)^7^ constructs a representation of biological events leading to adverse effects, and it provides a coarse granularity of events. However, an AOP mainly focuses on measurable changes (key events). In this study, we modelled a representation framework that describes toxic courses from latent to toxic manifestations. We represent a toxic course as causal relationships between toxic processes and introduce a model of the imbalance between biological defence functioning processes and toxic action processes. As a case study, we describe phospholipidosis. Because the imbalance model can represent the adaptation stage, it will contribute to early drug risk management by enabling an appropriate subpopulation of patients to be identified.

## Results

### Development of the TXPO for toxic mechanism knowledge systematization

#### Outline of the TXPO

**Figure 1:**
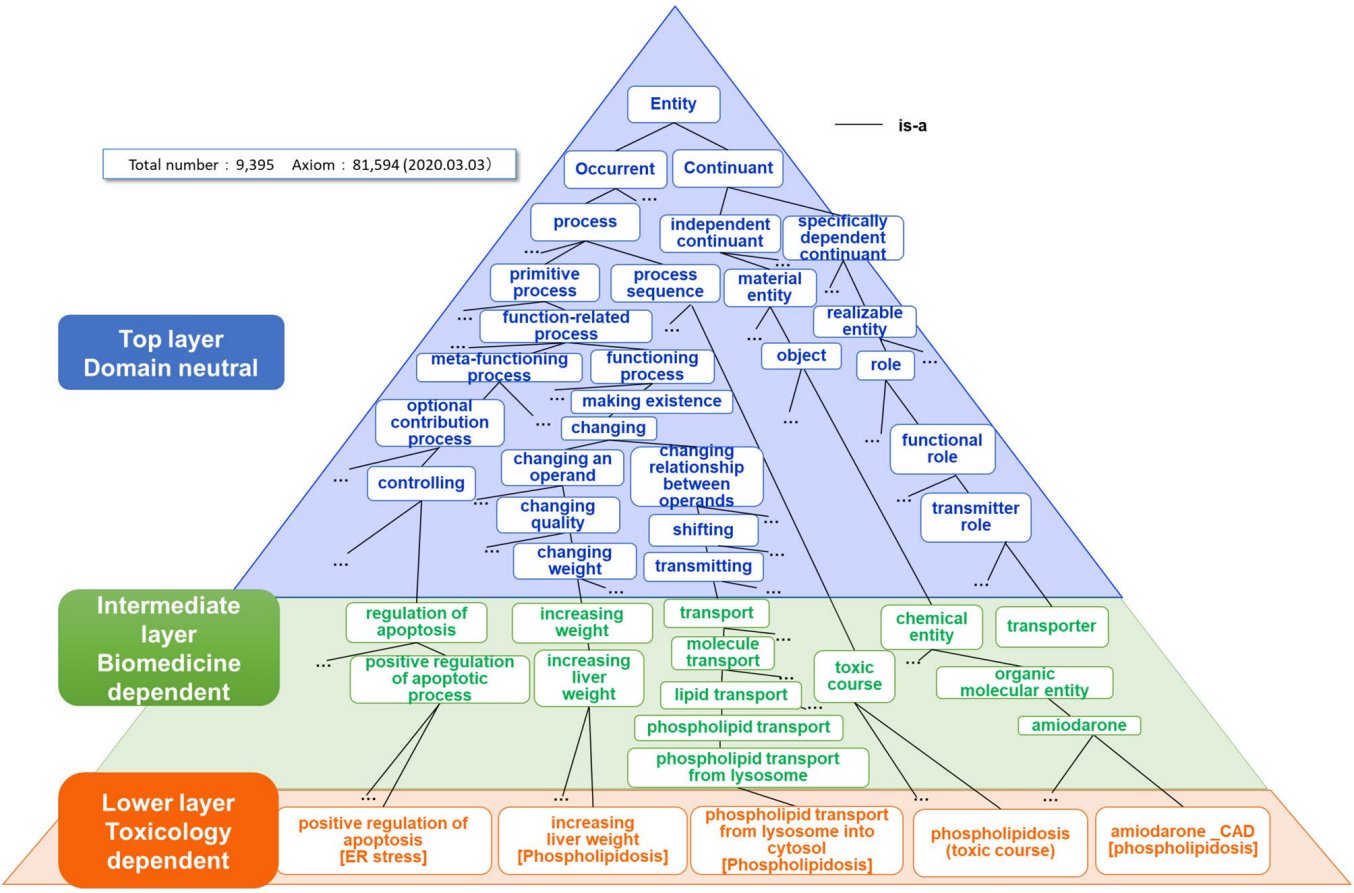
Overview of the ToXic Process Ontology (TXPO), which contains a three-layer *is-a* hierarchy: the top layer contains general terms, mostly from the Basic Formal Ontology (BFO). The intermediate and lower layers contain biomedical terms and more granular toxicology terms, respectively.

The TXPO is a three-layer model organized in an *is-a* hierarchy from general terms to specialized toxicological terms (Fig. 1). The top layer is domain-independent and provides general terms from the upper-level Basic Formal Ontology (BFO)^2^. BFO supports generic categories and relations based on a philosophical orientation. Accordingly, we constructed our ontology using the inheritance of the intrinsic nature of an entity in a consistent manner. All entities of the TXPO are classified as *continuant* or *occurrent*. The term *continuant* refers to an entity that persists, endures, or continues to exist through time while maintaining its identity and includes *objects*, *roles*, and *qualities*. An *object* is an independent continuant, such as a thing. *Roles* and *qualities* are dependent *continuants* that can only exist depending on something else. *Occurrent* includes entities that unfold over time, such as *processes*.

The intermediate layer consists of biomedical entities. These terms include anatomic structures from Uber-anatomy ontology (UBERON)^8^, cells from the Cell Ontology^9^, organisms from the NCBI Taxonomy^10^, compounds from Chemical Entities of Biological Interest (ChEBI)^11^, biological processes and cellular components from the Gene Ontology (GO)^12^, qualities from the Phenotype And Trait Ontology (PATO)^13^, some molecule families from INOH^14^, genes from the Ontology of Genes and Genomes (OGG)^15^, and diseases from the Disease Ontology^16^. The size of each ontology is vast, and many parts are unrelated to toxic mechanisms. Therefore, only those terms relevant to the toxic courses were manually imported.

The lower layer consists of entities specific to toxicology.

#### Process in the TXPO

*Process* is a central category in the TXPO. To elucidate a toxicity mechanism adequately, we provide two sub-categories: *primitive process* (a process that is a single unit) and *process sequence* (a series of processes, which includes pathways and toxic courses).

1. Functioning processes

**Figure 2:**
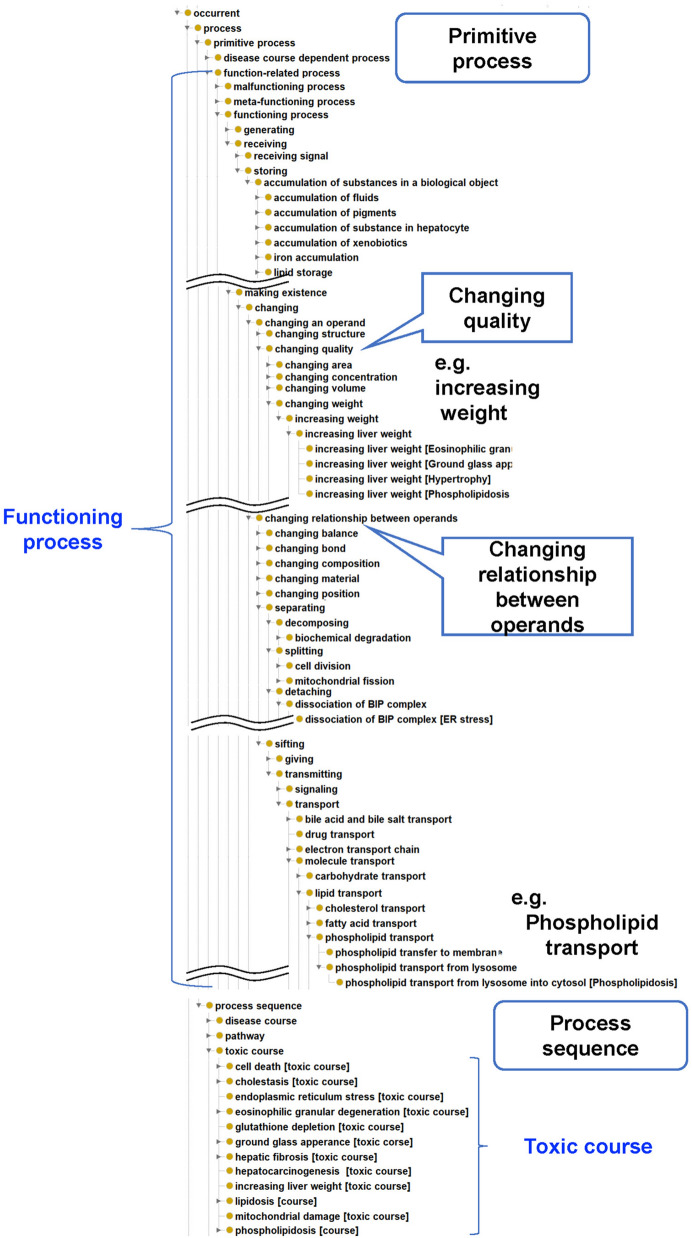
TXPO functioning process *is-a* hierarchy.

The function of many biological defence processes is to protect organisms from toxicity-associated injury. Therefore, we focus on functioning processes. Functioning processes in organisms vary in granularity, from the molecular level to the organelle, cell, tissue, and organ level. To define functioning processes consistently, we refer to functional ontology^17^. Functional ontology defines general functions based on changes in the state of the input–output relationship among physical things and models functional knowledge. The subclasses of *functioning process* in the TXPO top layer are imported and reused from functional ontology (Fig. 2). In detail, a *functioning process* can be mainly categorized into *receiving*, *making existence*, and *generating* categories. The *making existence* category can be further subdivided into *changing an operand* and *changing relationship between operands* classifications. *Changing an operand* includes changing qualities such as concentration, volume, or weight. Examples of subtypes of *changing relationship between operands* are *sifting* and *separating*. Subtypes of *sifting* include *transmitting*, and subtypes of *separating* include *decomposing*, *splitting*, and *detaching*. By specializing the entities in the top layer, we define original terms in the intermediate and lower layers in the TXPO. The intermediate layer is biomedical-domain dependent. For instance, a lower level of *transmitting* includes biological transport processes such as lipid transport. *Decomposing* includes biochemical degradation; *splitting* includes cell division; and *detaching* includes complex dissociation. Some entities can be mapped to GO biological processes, as some of GO terms can be interpreted as functional processes common to biomedicine.

The lower layer is toxicology-domain dependent. In this study, we define a ‘toxic process’ as a process that constitutes a specific toxic course. For example, to specialize the ‘phospholipid transport (GO: 0015914)’ in GO, we define an ‘phospholipid transport from lysosome into cytosol [Phospholipidosis]’ that constitutes a course of phospholipidosis.

One difficulty in defining a toxic process is that protective responses to drugs can also injure cells. Hence, to understand the toxicity mechanisms appropriately, we also regard a process that functions as a biological defence in the toxic course as a ‘toxic process’.

The development of the toxicity-dependent process subtree was based on the ‘low-hanging fruit’ policy. Terms were extracted and manually annotated from toxicology-related textbooks and articles.

In addition to the function-execution process, the TXPO defines *meta-functioning processes* as function-related processes specific to other functions and includes *controlling*, for example. Subtypes of *controlling* include the regulation of apoptosis and cell cycle control.

2. Decomposition of functioning

**Figure 3:**
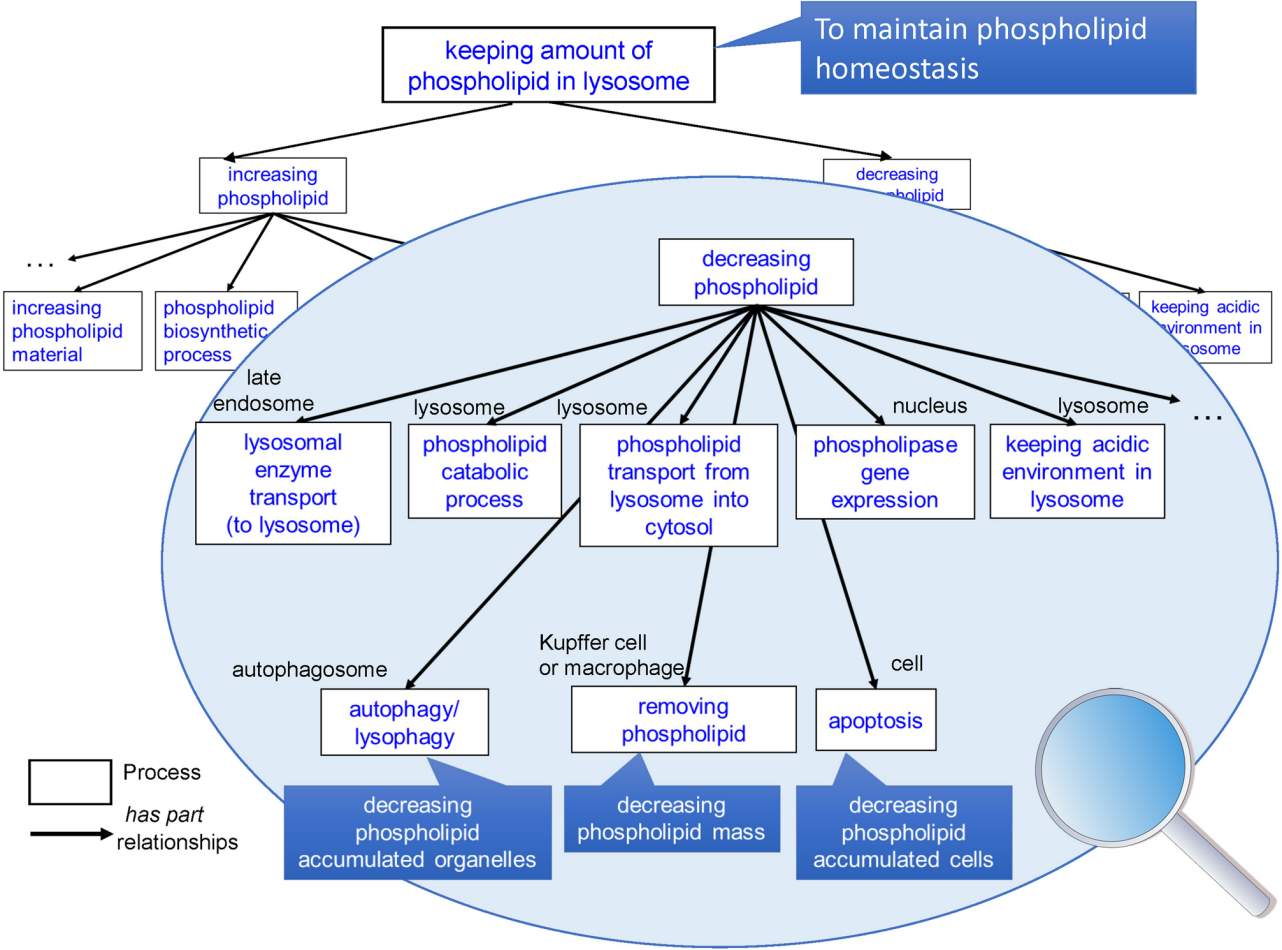
Example of a functional decomposition of ‘decreasing phospholipid’.

The TXPO specifies a functioning process based on a function decomposition framework. In an ontological engineering approach, a device (system) consists of sub-devices (sub-systems). In a function decomposition tree, the overall function of a system is achieved by a sequence of sub-functions of the sub-systems. Because biological functions can be considered specializations of systemic functions^18^, we attempted here to clarify the functioning process of biological structures for each granularity based on the whole–part relationship (part-of/has-part relationship). At the cell level, we regard a cell as a system and cell components such as organelles as system parts. Figure 3 shows an example describing how the cell system decreases phospholipids to maintain phospholipid homeostasis. Under normal conditions, the cell system maintains the phospholipid level by phospholipid import/export, phospholipid biosynthesis (anabolism)/catabolism, and similar processes. In the course of phospholipidosis, it is known that cationic amphiphilic drugs (CADs) bind to the lysosomal membrane, which triggers a negative regulation of phospholipid degradation, resulting in the accumulation of phospholipids in lysosomes. Therefore, the cell system performs a ‘decreasing phospholipid’ process as a biological defence function (enlarged part of Fig. 3). In the cell system, the organelle performs a sub-function as a sub-part. For example, ‘phospholipid catabolic process’ and ‘phospholipid transport from lysosome into cytosol’ work in lysosomes, and ‘lysosomal enzyme transport’ executes in the late endosome. Moreover, in the nucleus, a function that promotes degradation, ‘phospholipase gene expression’ is performed. Accordingly, the cell system reduces phospholipids by performing sub-functioning processes of the system parts.

However, when the accumulation of phospholipid becomes more severe, autophagy works to remove phospholipid mass in lysosomes, and apoptosis also plays a defensive role in removing phospholipid-accumulated cells in the liver. In this way, the functional decomposition approach can specify a variety of defence functions using whole-of-part relationships of a biological system within a unified representation framework. Furthermore, the function decomposition tree reveals that dysfunction in one process affects other processes from the bottom up. Therefore, this approach can help predict the progress of toxicity and drug risk management so that an alternative path can be found.

3. Toxic course definition

**Figure 4:**
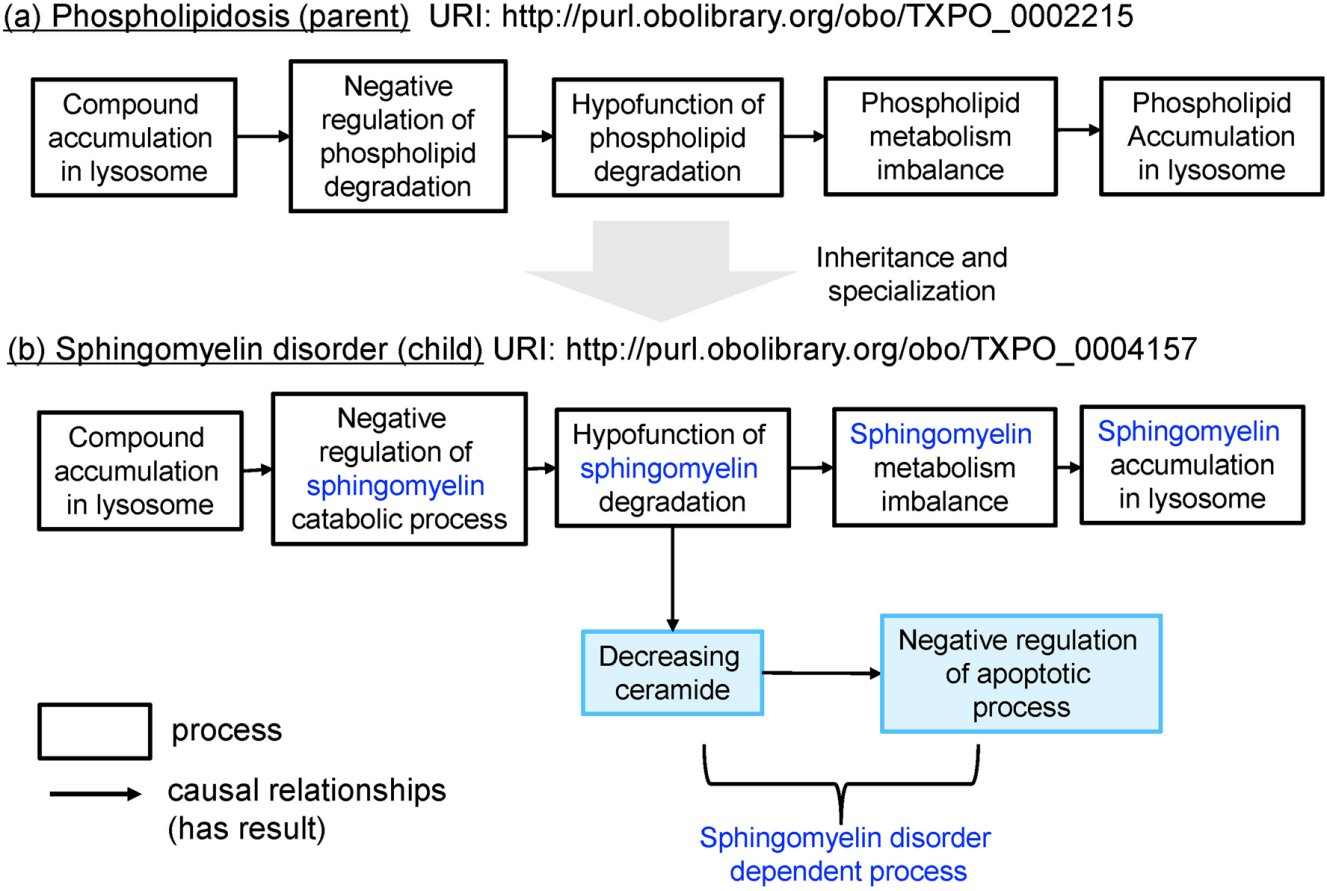
Examples of causal relationship representations of toxic courses: (**a**) phospholipidosis, (**b**) sphingomyelin disorder. Blue text indicates the specialization from phospholipid to sphingomyelin; blue boxes show additional sphingomyelin-dependent processes.

In the domain of toxicology, understanding a toxicity mechanism is crucial for drug safety management. Toxicity mechanisms are generally explained in terms of multiple processes such as toxicant delivery, biological defence process, cellular dysfunction/dysregulation, and cell death^1^. Therefore, to clarify the toxicity mechanism, we define a toxic course as a series of process in an organism that manifests toxicities and that are not part of the life of the organism. Processes in one toxic course are represented by the causal relationships between processes. Furthermore, the processes of a parent toxic course are inherited by the child toxic courses (Supplementary Information 1). Figure 4 illustrates an example of the causal relationships of phospholipidosis and the child toxic course sphingomyelin disorder. Drugs like CADs are known to negatively regulate phospholipid degradation in lysosomes^19^. In this study, phospholipidosis includes the following causal (has-result) relationships, indicated using ‘→’ (Fig. 4a):

Compound accumulation in lysosome

- → negative regulation of phospholipid degradation
- → hypofunction of phospholipid degradation
- → phospholipid metabolism imbalance
- → phospholipid accumulation in lysosome

All instances of sphingomyelin disorder are also instances of the parent toxic course, phospholipidosis. They include the causal relationships of the processes in phospholipidosis. Moreover, by specializing phospholipid into sphingomyelin, the course of sphingomyelin disorder holds the following causal relationships (Fig. 4b):

Compound accumulation in lysosomes

- → negative regulation of sphingomyelin catabolic process
- → hypofunction of sphingomyelin degradation
- → sphingomyelin metabolism imbalance
- → sphingomyelin accumulation in lysosome

In TXPO, a parent course can also be specialized into a child course by adding specific processes. For example, ‘hypofunction of sphingomyelin degradation’ can cause the following processes specific to the course of sphingomyelin disorder:

Hypofunction of sphingomyelin degradation

- → decreasing ceramide
- → negative regulation of apoptotic process

The toxic course currently contains more than ten courses on the following themes: ER stress, glutathione depletion, phospholipidosis, lipidosis, mitochondrial damage, ground glass appearance, eosinophilic granular degeneration, hepatocarcinogenesis, cell death, and increasing liver weight.

#### Role

In general, a molecule plays multiple roles in the body. Therefore, our goal was to explicate the roles of molecules participating in specific processes in the toxic course. For example, PLA2G15 (phospholipase A2 group XV) can act as a ‘positive regulator of immune response’ in the inflammatory response in severe phospholipidosis. As for drugs, TXPO makes their roles explicit in a specific toxic process. For example, amiodarone plays the roles of a 'cationic amphiphilic drug' and ‘competitive inhibitor of phospholipase’ and participates in the negative regulation of phospholipid degradation in phospholipidosis. Amiodarone also takes on a 'mitochondrial respiratory-chain inhibitor' role in the negative regulation of the respiratory electron transport chain in lipidosis.

#### Relationships among entities

**Figure 5:**
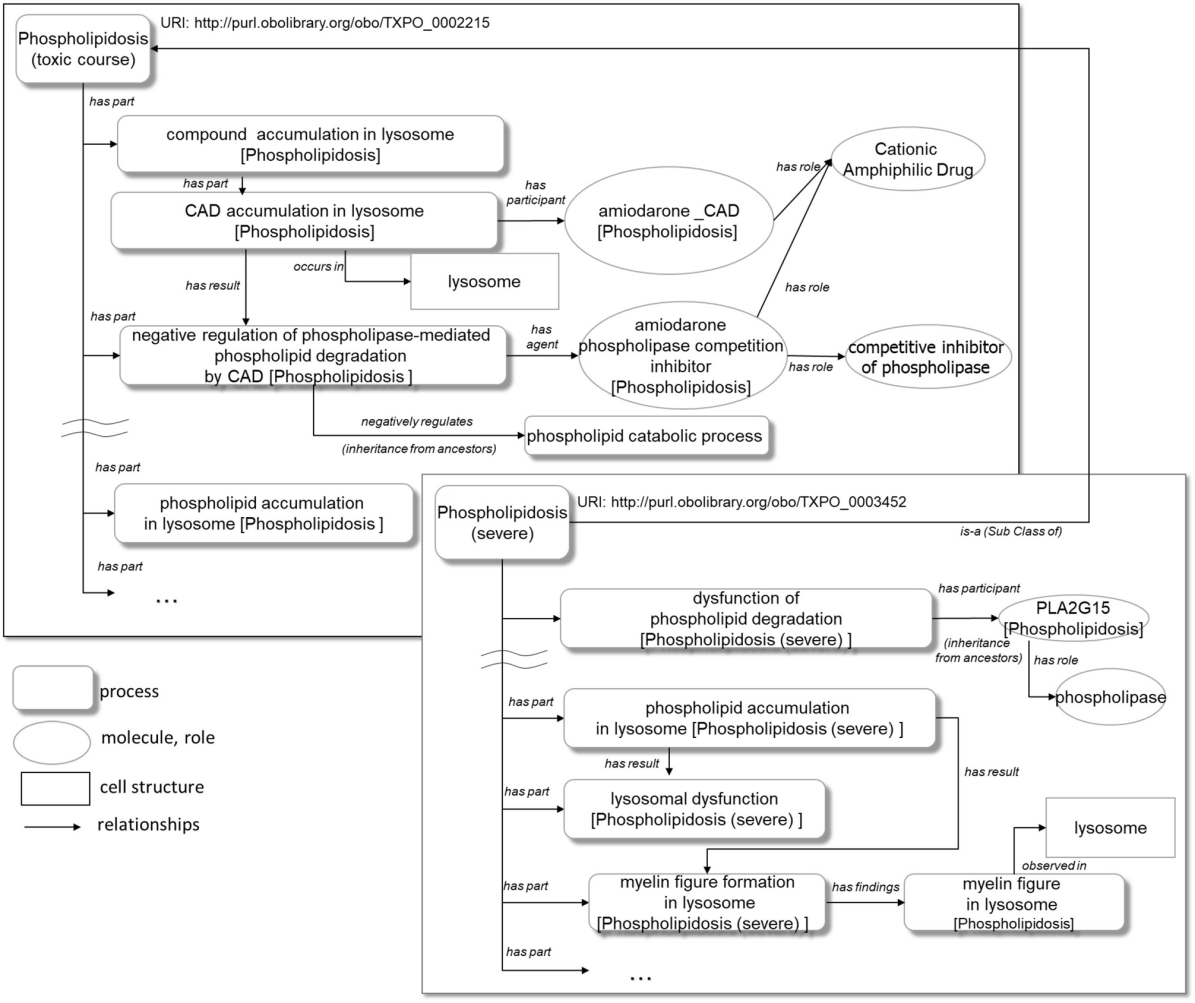
Examples of TXPO relationships. Rounded rectangles indicate toxic courses and processes; ellipses indicate molecules and their roles. Boxes represent biological structures, e.g. lysosome. Not all processes are shown. In the phospholipidosis toxic course, for instance, CAD accumulation in lysosome process *occurs in* lysosome and amiodarone *has role* cationic amphiphilic drug.

As of March 3, 2020, 9,395 entities have been defined in the TXPO. Figure 5 shows an example of the relationships among the terms defined in phospholipidosis.

### Toxic course map as a representation framework for early risk management of drugs

To address drug safety management from an early stage, it is important to represent the processes before the onset of toxicity for computer analysis. Cells constantly adapt to physiological demand to maintain a homeostatic steady state^20^, and adaptation can be thought as a process to maintain homeostasis against functional demand. In this study, we introduce a demand and supply imbalance model^21^ into the toxic course representation. In this model, supply indicates the functioning processes associated with biological defence and demand refers to toxic activity. The basic units are as follows (Supplementary Information 2):

1. a functioning process (supply) as a biological defence for maintaining homeostasis;
2. a functional demand process (demand) as toxic activity;
3. balance/imbalance between toxic activity and defence processes;
4. outcome of the organelles, cells, or tissues of the organ exhibiting toxicity manifestations.

The level of functioning performance can change according to changes in demand; however, if demand exceeds performance, an imbalance and outcomes that manifest toxicity occur.

To understand the precise mechanisms in idiosyncratic toxicity, the levels of functional performance must be considered. Here, we introduce the following levels: ‘very low’, ‘low’*, ‘*medium’*, ‘*high’, and ‘very high’. Since cells normally maintain homeostasis in which the milieu is maintained within a narrow range^22^, we define this level of functioning as ‘medium’. In the body’s system, if the functional demand increases to a high level, the defence function also performs at a high level to adapt, and a new homeostasis is obtained. However, under severe stimulus conditions, e.g. drug exposure, a ‘very high’ toxic action results in an imbalance and can lead to irreversible cell injury and cell death. When a ‘very high’ level of defence function exceeds demand, it can also lead to serious damage such as liver fibrosis (details are given in Supplementary Information 2).

#### Imbalance model in phospholipidosis

**Figure 6:**
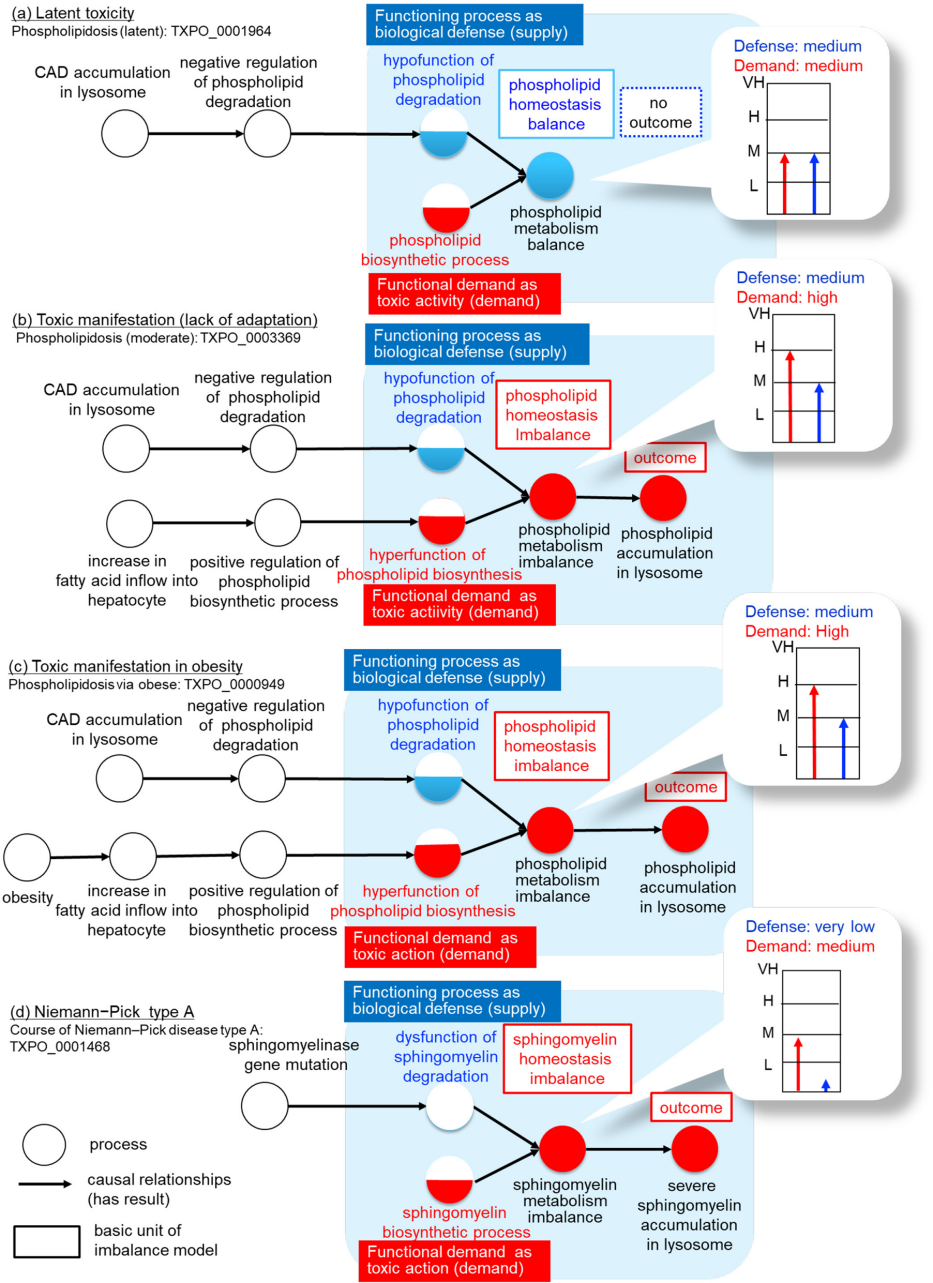
Imbalance model for phospholipidosis. Circles indicate processes; links between nodes indicate causal relationships (has result relationships). Boxes represent the basic units of the model: a functioning process (supply) as a biological defence, a functional demand process (demand) as toxic activity, balance/imbalance, and outcome. Not all processes are shown. (**a**) Latent toxicity (Phospholipidosis (latent): http://purl.obolibrary.org/obo/TXPO_0001964): although balance is maintained, the body system cannot adapt when CADs are present. (**b**) Toxicity manifestation (Phospholipidosis (moderate): http://purl.obolibrary.org/obo/TXPO_0003369): While CAD accumulation causes the negative regulation of degradation, other causes, e.g. positive regulation of phospholipid biosynthesis, cause hyperfunction of phospholipid biosynthesis, causing imbalance and phospholipid accumulation as an outcome. (**c**) Toxic manifestation in obesity (Phospholipidosis via obese: http://purl.obolibrary.org/obo/TXPO_0000949). (**d**) Niemann–Pick type A (course of Niemann–Pick disease type A: http://purl.obolibrary.org/obo/TXPO_0001468).

Here, we applied the imbalance model to an example of a toxic course, phospholipidosis. In phospholipidosis, the basic units are (1) increasing the phospholipid process as functional demand to decrease phospholipid, (2) decreasing the phospholipid process as a defence function, (3) phospholipid homeostasis balance/imbalance between functional demand and function, and (4) the ‘phospholipid accumulation’ process as an outcome. In the drug exposure situation in phospholipidosis, it is known that CADs affect biological functioning. CADs bind to lysosomal membranes and form a complex with phospholipids, which negatively regulates phospholipid degradation^19^. Under normal conditions, the cell system can maintain a balance between ‘phospholipid biosynthetic process’ and ‘phospholipid degradation’ at a ‘medium’ level. However, when the CADs are not excreted from the body, the defence system cannot perform at a ‘high level’, i.e. there is hypofunction of phospholipid degradation. Although no outcomes occur, the cellular system is in the so-called latent stage (Fig. 6a) Here, if the functional demand is increased by additional causes such as ‘positive regulation of phospholipid biosynthetic process’ caused by an ‘increase in fatty acid inflow into hepatocyte’, then an imbalance can occur. As a result, ‘phospholipid accumulation in lysosome’ manifests as an outcome, i.e. there is a lack of adaptation (Fig. 6b) For example, obesity causes an ‘increase in fatty acid inflow into hepatocyte’, which can promote phospholipid biosynthesis (Fig. 6c). Therefore, obese patients with chronic CAD use could be disposed to have a phospholipid homeostasis imbalance.

In Niemann–Pick disease type A, because of a ‘sphingomyelinase gene mutation’, ‘dysfunction of sphingomyelin degradation’ can occur, which decreases the defence level to ‘very low’ (Fig. 6d). Therefore, an imbalance always occurs regardless of the existence of CADs, and sphingomyelin accumulation is observed as an outcome. Several drugs such as tricyclic antidepressants (e.g. desipramine and imipramine) have been shown to induce functional loss of acid sphingomyelinase activity in vivo^23^. Desipramine protects hepatocellular apoptosis via the inhibition of ceramide channel formation^24^, so chronic use of imipramine might induce the accumulation of sphingomyelin, as in Niemann–Pick disease, and inhibit ceramide production.

In summary, there are various patterns of imbalance that can occur in a toxic course. In this study, we focused on hepatotoxicity. However, toxic expression varies depending on the organ function. For instance, in phospholipidosis in the lung, the function of pulmonary surfactant may be impaired, and phospholipidosis in the heart can lead to a life-threatening disorder such as QT prolongation due to cell hypertrophy caused by phospholipid accumulation. In future, we plan to analyse toxic courses in other organs.

### Application: development of the TOXPILOT knowledge system

One of the advantages of an ontology is it can assist navigation to appropriate knowledge, which is useful for various applications in drug safety evaluation and management. In this study, we developed a toxic process interpretable support knowledge system called TOXPILOT (https://toxpilot.nibiohn.go.jp). The TOXPILOT system provides useful information to help users acquire knowledge concerning toxic process based on the TXPO, which helps bridge the gaps between basic science and medicine and accelerate knowledge sharing across disciplines for drug safety management. Using semantic web technology, TOXPILOT supports the interpretation of toxicity mechanisms and visualizes toxic courses with useful information based on ontology. Retrospective and forward analyses from one common finding demonstrate how the processes of phospholipidosis and non-alcoholic steato-hepatitis (NASH) can be compared.

1. Toxic course map

**Figure 7:**
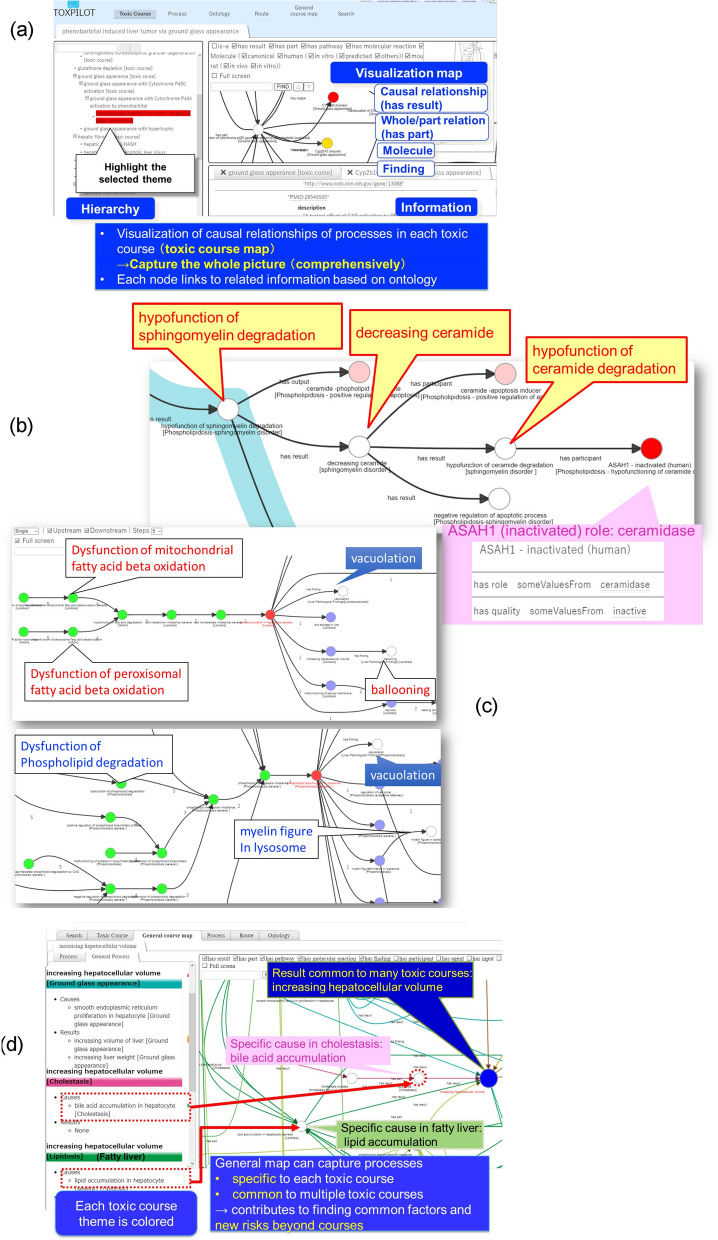
Toxic process interpretable knowledge system (TOXPILOT) (**a**) The toxic course map visualizes a toxic course as causal relationships between processes. (**b**) In vitro marker genes visualized in the phospholipidosis map. (**c**) Route search provides the upstream or downstream paths of the selected process. (Upper) Route search from vacuolation in phospholipidosis. (Lower) Route search from vacuolation in NASH. (**d**) The general course map visualizes general toxic courses common to multiple specific toxic courses (differentiated by colour).

TOXPILOT provides toxic course maps that can visualize the causal relationships of toxic processes, as described in the previous section (Fig. 7a). In a toxic course map, a toxic course is shown as a graph consisting of nodes representing processes and links representing their causal relationships. This map can also show the molecules that participate in the process and pathological findings.

Our preliminary data show that the maps enable us to visualize marker genes for liver toxicity predicted by support vector machines. We can hence identify genes that are predicted to participate in common processes in ER stress based on rat in vivo and human in vitro analyses^25,26^. Thus, this toxic course map facilitates evaluation and extrapolation to humans for translational research.

TOXPILOT also provides process maps based on a functional decomposition approach. These maps enable the visualization of pathologic findings associated with a process.

2. Support for an in vitro screening system

Next, we examined whether TOXPILOT can support the prediction of toxic marker genes in an in vitro screening system using large-scale DNA microarray gene expression analysis for phospholipidosis. We selected one article on the human in vitro gene prediction of phospholipidosis^27^ and annotated the marker genes predicted in the article with respect to related processes, molecule role, and causal relationships. Figure 7b shows the results. For example, for ASAH1 (N-acylsphingosine amidohydrolase 1) in the sphingomyelin disorder, which is a sub-type of phospholipidosis, our map shows that inactivated ASAH1 participates in the ‘hypofunction of ceramide degradation’ process. ASAH1 encodes the enzyme, acid ceramidase. In sphingomyelin metabolism, sphingomyelin degradation produces ceramides, and acid ceramidase catalyses ceramide degradation. However, in the sphingomyelin disorder, ‘hypofunction of sphingomyelin degradation’ can cause ‘decreasing ceramide’, which might change acid ceramidase coding gene ASAH1 expression, and ‘hypofunction of ceramide degradation’ can occur. TOXPILOT also provides access to a wealth of information about each node. For instance, ASAH1 plays a role in the ceramide signalling pathway and other processes such as inducing apoptosis for tumour cell death.

Hence, for in vitro screen analysis, it is possible for this system to show marker genes and their roles in related processes according to the mechanisms. In general, genes are known to play multiple roles in the body, and TOXPILOT shows the role of each gene in the specific process according to the mechanism, which contributes to drug safety evaluation.

3. Route search

In the development of pharmaceuticals, the requirements for a drug safety assessment system will differ depending on the development stage. In the early stages of drug discovery, developers want to know how the process will progress and whether a serious disorder will occur in the body. In contrast, in the pre-clinical stage, pathologists prefer to obtain information about the possible causes of an effect before observing the pathological findings.

The TOXPILOT system can provide both forward and backward route searches from a node of interest in a toxic course map. Figure 7c shows an example that compares the differences in retrospective analyses of lipid vacuolation as a pathological finding for phospholipidosis and NASH. In the course of NASH, vacuolation involves lipid accumulation, and the upstream process is dysfunction of fatty acid degradation and the like (Fig. 7c, upper part). In contrast, in the course of phospholipidosis, the vacuolation involves phospholipid accumulation due to phospholipid metabolism imbalance caused by dysfunction of phospholipid degradation, upstream of which is negative regulation of phospholipase-mediated phospholipid degradation caused by CAD (Fig. 7c lower part). Next, when comparing the route of downstream processes in each toxic course, the findings for NASH reveal that increasing hepatocellular volume involves ballooning, whereas in phospholipidosis, myelin figure in lysosome can be shown in each toxic course. Therefore, users can capture the differences in the specific pathological findings of each course. Hence, our map not only helps users search for the route of each toxic course, but it also helps them understand the differences between toxic courses given one common process or finding.

4. General course map

One problem in drug safety research is how to reduce unexpected toxicity. Therefore, it is important to provide comprehensive information about the causal relationship of mechanisms across multiple toxic courses. The TXPO provides a general course map that visualizes general toxic courses common to multiple specific toxic courses based on ontology (Fig. 7d). In drug safety evaluation, toxicologists sometimes want to know whether one phenomenon occurring in a particular toxic course could occur in other toxic courses. For instance, in the course of phospholipidosis, ‘phospholipid accumulation in lysosome’ can cause ‘increasing hepatocyte volume’. The TXPO system extracts information from the Resource Description Framework (RDF) database using SPARQL and automatically generates a general course map (Supplementary Information 4). In this map, common processes are represented as large nodes. Users can see that ‘increasing hepatocyte volume’ is common to other toxic courses such as cholestasis. Moreover, users can obtain information regarding different causes associated with other courses. Here, it is clear that ‘bile acid accumulation’ occurs specifically in the course of cholestasis and in other courses such as ‘smooth endoplasmic reticulum proliferation’ in ground glass appearance’.

Furthermore, by generalizing causes using the ontological hierarchy tree, the possible causes of increasing cell volume such as increases in the number of organelles or accumulation of intracellular substances can be classified. Cells have very specialized functions under normal conditions; however, in a toxic environment, they might increase their protective function through alternative pathways or perform alternative functions. By analysing both general and specific causal relationships, theoretically possible mechanisms for new paths could be found that could not be obtained by each course theme, which would improve drug risk discovery for drug safety evaluation.

## Discussion

Concerning toxicity, there are many databases^28^ such as DrugMatrix (https://ntp.niehs.nih.gov/drugmatrix/index.html) and the Liver Toxicity Knowledge Base (LTKB)^29^, however, they focus on genomes or compounds. By employing systemic functional decomposition, the TXPO covers various processes across granularities in a consistent manner. We confirmed that we can describe both pathway- and molecular-level processes in a unified manner regarding ER stress. However, we found that the number of molecular processes is so large, it can be difficult to grasp the overall picture of the mechanism. Therefore, the TXPO deals primarily with process–process interactions with granularities starting from the organelle level. At the molecular level, we describe molecules as participants in toxic course processes. Furthermore, we explain the role of each molecule in a given specific process. Regarding in vitro screening, TOXPILOT can visualize marker genes and explicate the molecule roles in a toxic course, which will contribute to early risk management during drug discovery.

Understanding toxicity mechanisms is challenging. Among the many issues involved, one aspect is the complexity of various interactions in a toxic course. We demonstrated that our imbalance model can clarify the context and distinguish toxic actions from body defence functions at each granularity, thus facilitating the interpretations of toxic mechanisms.

In general, expert knowledge is hard for other experts to understand; for example, molecular biologists find it extremely difficult to distinguish findings in pathology, and vice versa, which fragments knowledge and makes it difficult to capture an overall picture of toxicities. The Comparative Toxicogenomics Database (CTD)^30^ can be used to provide pathways and processes such as KEGG and GO. However, because each piece of information is provided independently, users cannot perceive the causal relationships of toxic processes. TXPO can provide systematized flexible information about molecules, pathways, and toxic processes through generalization, specialization, and other relationships in a consistent manner. Based on a philosophical view, ontology makes the intrinsic nature explicit. Using the systematized knowledge infrastructure of TXPO, TOXPILOT supports the interpretation of toxicity mechanisms, accelerates knowledge sharing, and realizes knowledge interoperability.

TXPO and TOXPILOT also help bridge the gap between basic science and clinical medicine. For example, ICD11 (https://icd.who.int/browse11/l-m/en) and Disease Ontology^16^ do not include the term ‘phospholipidosis’. However, our ontology reveals that the sphingomyelin disorder, which is a type of phospholipidosis in toxicology, and Niemann–Pick types A and B have the following commonalities in their related processes:

- Structural commonality: lysosomes
- Causal relationship commonality: dysfunction of sphingomyelin degradation causes sphingomyelin accumulation in lysosomes
- Finding commonality: macrophage aggregation and myelin figures as myelin-like layered structures under electron microscopy

Regardless of whether they are internal or external factors, our system can provide causal relationships owing to dysfunction in the body system, homeostasis imbalance, and its outcomes. Our approach is hence able to transfer knowledge between toxicology and clinical medicine through commonalities. Therefore, from the perspectives of both toxicity and disease, TOXPILOT can support both drug risk management in drug development and therapeutic management in medicine. The imbalance model could determine a functioning performance level, which could help select patient groups with potential idiosyncratic toxicities. Because it includes latent toxicity, TOXPILOT will be useful for supporting patient care as a therapeutic approach in precision medicine. In this way, TOXPILOT enables knowledge sharing and generates interdisciplinary knowledge cycles based on the TXPO, and this could help elucidate complex mechanisms of toxicity.

We are currently annotating more toxic courses and enhancing the level of sophistication of the terms in the TXPO. In future, we plan to cover toxic courses in other organs. Recently, the TXPO joined the Open Biological and Biomedical Ontology (OBO) Foundry for knowledge sharing among not only toxicologists but also other biomedical communities. Via the OBO Foundry site (http://www.obofoundry.org), TXPO is available from the NCBO BioPortal, Aber-OWL (http://aber-owl.net/ontology/TXPO) and Ontobee (http://www.ontobee.org/browser/index.php?o=txpo).

A limitation of our basic principle of focusing on toxic processes is that we do not have much information about drug efficacy. To increase the comprehensiveness of knowledge in drug development, it may necessary to collaborate with other ontologies such as the Drug Target Ontology^31^. We are now extending valuable knowledge from various other ontologies, such as the Disease Ontology and Human Phenotype Ontology^32^. Bridging the gap in toxicity knowledge from the domains of basic science to clinical medicine could help elucidate multiple mechanisms of toxicity.

## Methods

### TXPO development

Using textbooks^1,20,33–35^, we researched drug-induced hepatotoxic mechanisms and obtained information about toxic courses and related processes, molecules (and their roles), and biological structures. Next, we searched for the latest information from toxic course-related articles using the PubMed search terms listed in Supplementary Table S1.

We used the ontology editing tool Protégé 5.2.0^36^ to develop the TXPO in the Web Ontology Language (OWL) and HermiT reasoner^37^, which is a Protégé plug-in.

Supplementary Schema S1 shows examples of the TXPO development process, which consisted of the following steps. (1) Each toxic course was defined, and related information was annotated using the Annotation Properties. (2) The processes constituting each toxic course were described using a ‘has part’ relation as an Object Property. (3) Each process was generalized using an *is-a* hierarchy to reveal processes common to multiple toxic courses, biological processes, and biomedical-independent processes. (4) Each process was decomposed into sub-processes (using the ‘has part’ relation). (5) The biological structure in which the process takes place was described (using ‘occurs in’). (6) Molecules, drugs, and their roles in the process were defined. (7) Causal relationships between process were defined using a ‘has result’ relation.

When generalizing the *is-a* tree structure, we reused existing ontologies. Domain-independent general entities were based on BFO, and biomedical entities were imported manually from existing ontologies from the NCBO BioPortal^38^. These biomedical ontologies include UBERON^8^, Cell Ontology^9^, NCBI Taxonomy^10^, ChEBI^11^, Gene Ontology^12^, PATO^13^, INOH^14^, and the OGG^15^.

### TOXPILOT development

TOXPILOT consists of an ontology library, an RDF database, and a web application system (Supplementary Fig. S1). The TXPO file is stored in the ontology library, and the file is converted into RDF format, which represents data using a triplet of Subject, Predicate, and Object, by Protégé. The RDF data are then stored in an RDF store using Apache Jena Fuseki (https://jena.apache.org/documentation/fuseki2/) and a SPARQL endpoint is constructed. For the web application system for TOXPILOT, the necessary information is dynamically acquired via SPARQL queries. Moreover, TOXPILOT generates graphs using D3.js^39^, which is a JavaScript library. TOXPILOT is publicly available online (https://toxpilot.nibiohn.go.jp). New term requests and issue reporting can be made via its GitHub tracker (https://github.com/txpo-ontology/TXPO/issues).

## Supplementary information

Supplementary information.
