# Supplementary material for: Ontological approach to the knowledge systematization of a toxic process and toxic course representation framework for early drug risk management
Source: Sci Rep. 2020 Sep 3;10:14581. doi: 10.1038/s41598-020-71370-7 (PMC7471325; doi:10.1038/s41598-020-71370-7)
Supplement: Supplementary file 1 — Supplementary information. [file 41598_2020_71370_MOESM1_ESM.pdf]

## Title

Ontological approach to the knowledge systematization of a toxic process and toxic course representation framework for early risk management


## Authors

Yuki Yamagata[1,2*] and Hiroshi Yamada[1*]

## Institutions and Addresses

[1] Toxicogenomics Informatics Project, National Institute of Biomedical Innovation, Health and Nutrition, 7-6-8 Saito-Asagi, Ibaraki, Osaka 567-0085, Japan

[2] Laboratory for Developmental Dynamics, RIKEN Center for Biosystems Dynamics Research, 2-2-3 Minatojima-minamimachi, Chuo-ku, Kobe 650-0047, Japan

## Correspondence:

Yuki Yamagata (E-mail: yuki.yamagata@riken.jp)
Hiroshi Yamada (E-mail: h-yamada@nibiohn.go.jp)


**Supplementary Information 1**

Toxic course definition and causal relationship representation

**Supplementary Information 2**

Toxic imbalance model representation framework

**Supplementary Information 3**

Figures, Table and Schema

**Supplementary Information 4**

SPARQL query examples

**Supplementary Information 1.** Toxic course definition and causal relationship representation

In this study, we define toxic courses in TXPO using Protégé 5.2. As described in Methods, the processes specified in each toxic course are shown using ‘has part’ relation of Object Property. The processes of parent toxic course are inherited by the child toxic courses.

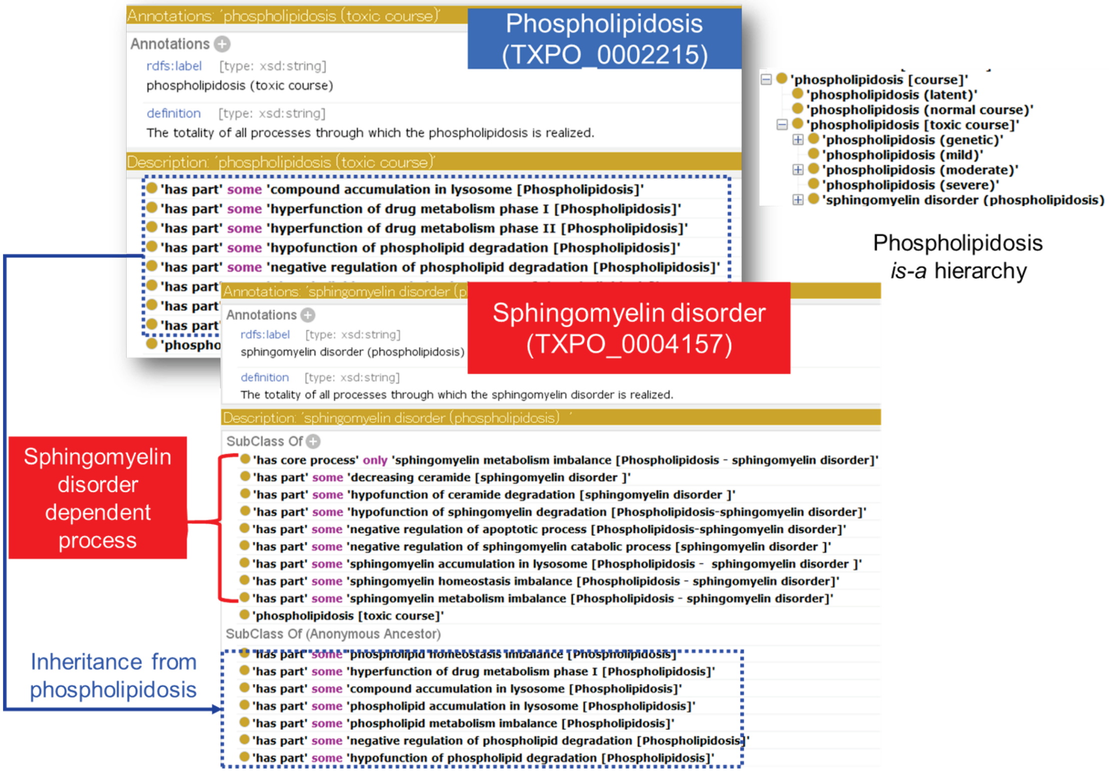


*Is-a* (subclass of) relationships of processes between toxic courses are represented as follows: For example, by inheriting and specializing ‘hypofunction of phospholipid degradation’ in the course of phospholipidosis, ‘hypofunction of sphingomyelin degradation’ in the course of sphingomyelin disorder is defined, which has context ‘sphingomyelin disorder’ with the constraint owl:allValuesFrom (shown as ‘only’ in Protégé.)

Each process has “has result” relations that describe the possible result process(es) in the toxic course. For example, ‘hypofunction of sphingomyelin degradation’ has the possible results ‘sphingomyelin metabolism imbalance’ and ‘decreasing ceramide’ in sphingomyelin disorder. The former represents an inherited and specialized process, and the latter represents a new process defined in the child course.

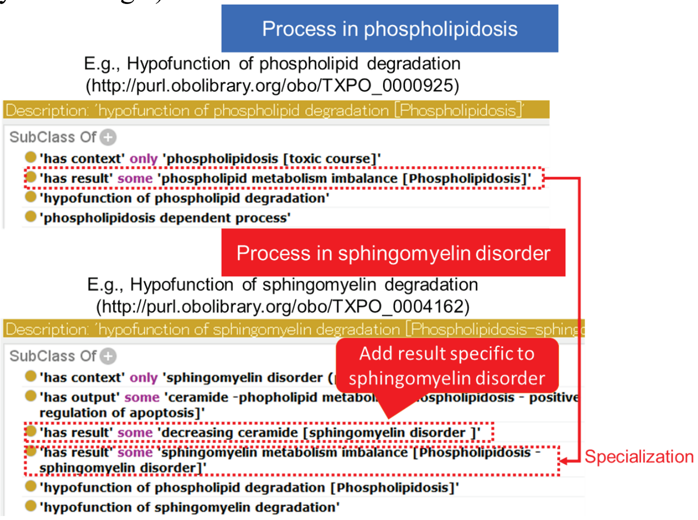

## **Supplementary Information 2.** Toxic imbalance model representation framework

In this imbalance model, we represent the basic units as four processes:
1) a functioning process (supply) as a biological defense for maintaining homeostasis;
2) a functional demand process (demand) as toxic activity;
3) balance/imbalance between toxic activity and defense processes;
4) outcome of the organelles, cells, or tissues of the organ exhibiting toxicity manifestations.

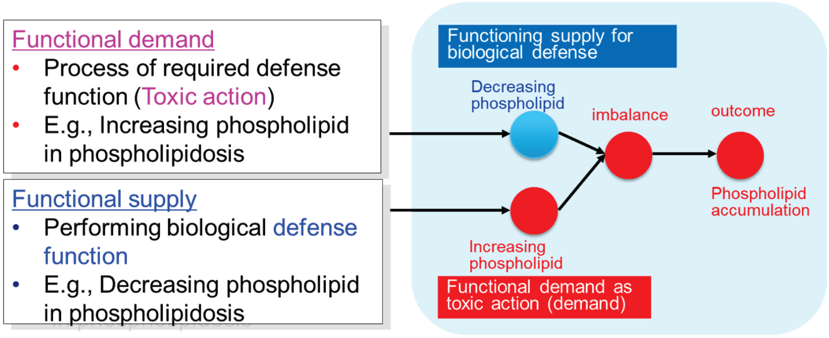


The level of functioning performance can change according to changes in demand; however, if demand exceeds performance, an imbalance and outcomes that manifest toxicity occur.
Here, we introduce the following levels: ‘very low’, ‘low’, ‘moderate’, ‘high’, and ‘very high’

1) Normal condition
Since cells normally maintain homeostasis in which the milieu is maintained within a narrow range, we define this level of functioning as ‘moderate’.

2) Adaptation
In the body system, if the functional demand increases from normal to a high level, the defense function also performs at a high level and achieves a new homeostasis as adaptation. During the adaptation, it is necessary to maintain homeostasis at a higher state than normal. Examples are hypertrophy and hyperplasia, both of which use processes functioning at a high level to meet increasing demands.

3) Toxicity manifestation from adaptation failures
Even if homeostasis is maintained at a higher level, when the demand increases to ‘very high’ by some additional factors. As a result, an imbalance can occur between the demand and defense function, which leads to cell injury and death as an outcome.

4) Drug-induced irreversible damage
Under severe stimulus conditions, a high level of drug exposure performs a ‘very high’ toxic action, which results in an imbalance between the demand and defense function. This results in irreversible cell injury and cell death.

5) Functional excess
Sometimes, the level of biological defense function exceeds demand and becomes ‘very high’. In that case, cell damage also occurs. Serious results such as tumor cell proliferation and fibrosis can occur.

6) Lack of adaptation
If the function level cannot be increased from ‘moderate’ to ‘high’ for some reason, the ability to adapt will degrade. Accordingly, in response to a stimulus, imbalance and cell damage can occur.

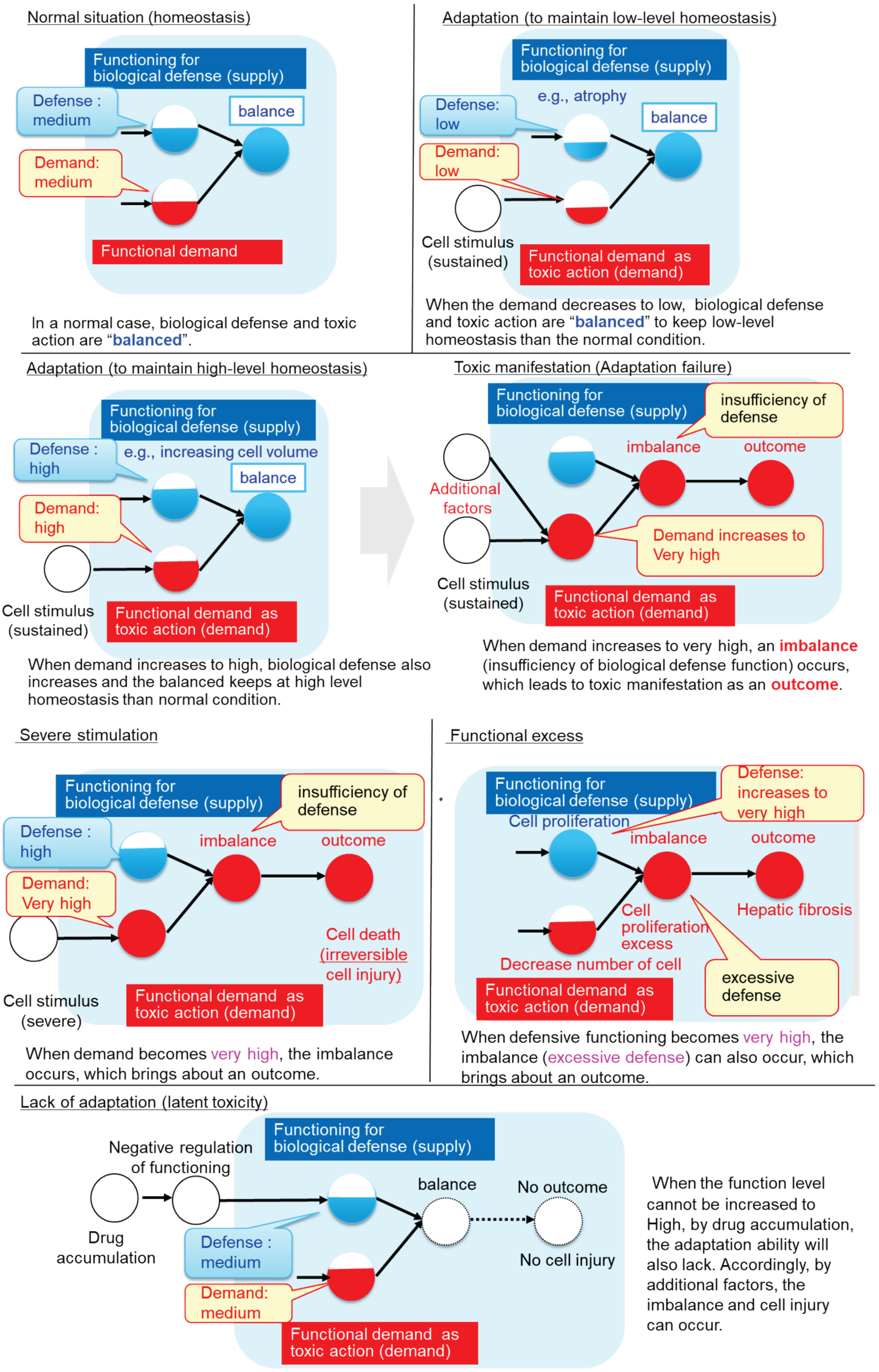

Normal situation (homeostasis)
Functioning for biological defense (supply)
Defense : medium
balance
Demand: medium
Functional demand
In a normal case, biological defense and toxic action are "balanced".
Adaptation (to maintain low-level homeostasis)
Functioning for biological defense (supply)
e.g., atrophy
Defense: low
balance
Demand: low
Cell stimulus (sustained)
Functional demand as toxic action (demand)
When the demand decreases to low, biological defense and toxic action are "balanced" to keep low-level homeostasis than the normal condition.
Adaptation (to maintain high-level homeostasis)
Functioning for biological defense (supply)
Defense : high
e.g., increasing cell volume
balance
Demand: high
Cell stimulus (sustained)
Functional demand as toxic action (demand)
When demand increases to high, biological defense also increases and the balanced keeps at high level homeostasis than normal condition.
Toxic manifestation (Adaptation failure)
Functioning for biological defense (supply)
insufficiency of defense
imbalance
outcome
Additional factors
Demand increases to Very high
Cell stimulus (sustained)
Functional demand as toxic action (demand)
When demand increases to very high, an imbalance (insufficiency of biological defense function) occurs, which leads to toxic manifestation as an outcome.
Severe stimulation
Functioning for biological defense (supply)
insufficiency of defense
Defense : high
imbalance
outcome
Demand: Very high
Cell death (irreversible cell injury)
Cell stimulus (severe)
Functional demand as toxic action (demand)
When demand becomes very high, the imbalance occurs, which brings about an outcome.
Functional excess
Functioning for biological defense (supply)
Defense: increases to very high
Cell proliferation
imbalance
outcome
Cell proliferation excess
Hepatic fibrosis
Decrease number of cell
excessive defense
Functional demand as toxic action (demand)
When defensive functioning becomes very high, the imbalance (excessive defense) can also occur, which brings about an outcome.
Lack of adaptation (latent toxicity)
Negative regulation of functioning
Functioning for biological defense (supply)
balance
No outcome
Drug accumulation
Defense : medium
No cell injury
Demand: medium
Functional demand as toxic action (demand)
When the function level cannot be increased to High, by drug accumulation, the adaptation ability will also lack. Accordingly, by additional factors, the imbalance and cell injury can occur.

We developed the computer representation framework of the toxic imbalance model and introduced degree levels for representing the functional demand/ supply in TXPO using Protégé 5.2. As illustrated in the original text, the degree of functioning performance can change according to changes in demand; however, if demand exceeds the performance of functioning, an imbalance occurs and results in outcomes that manifest toxicity.

An imbalance/balance process has one toxic composite imbalance state. The toxic composite imbalance state has 1) a biological defense functioning state and 2) a functional demand state, and Both of each defense (supply) and demand have a degree level.
The degree level is a subtype of ‘qualitative quantity’ and has five levels: Very low, small, low, high, and very high in the TXPO is-a hierarchy.

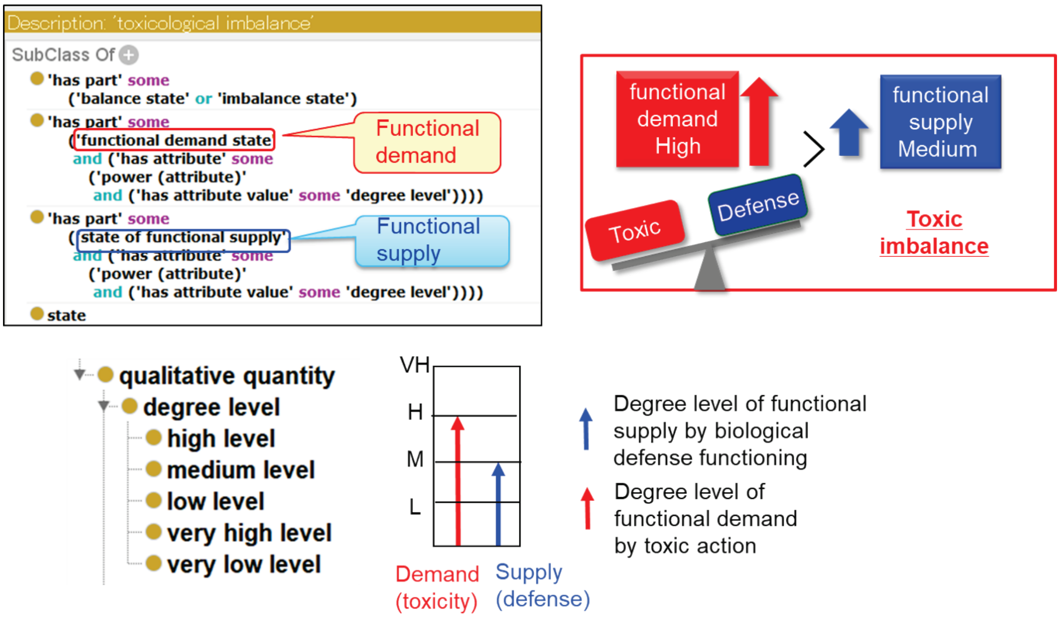


When the normal condition, the balance is keeping represented by ‘balance’ state supply and functional demand both at Medium levels.

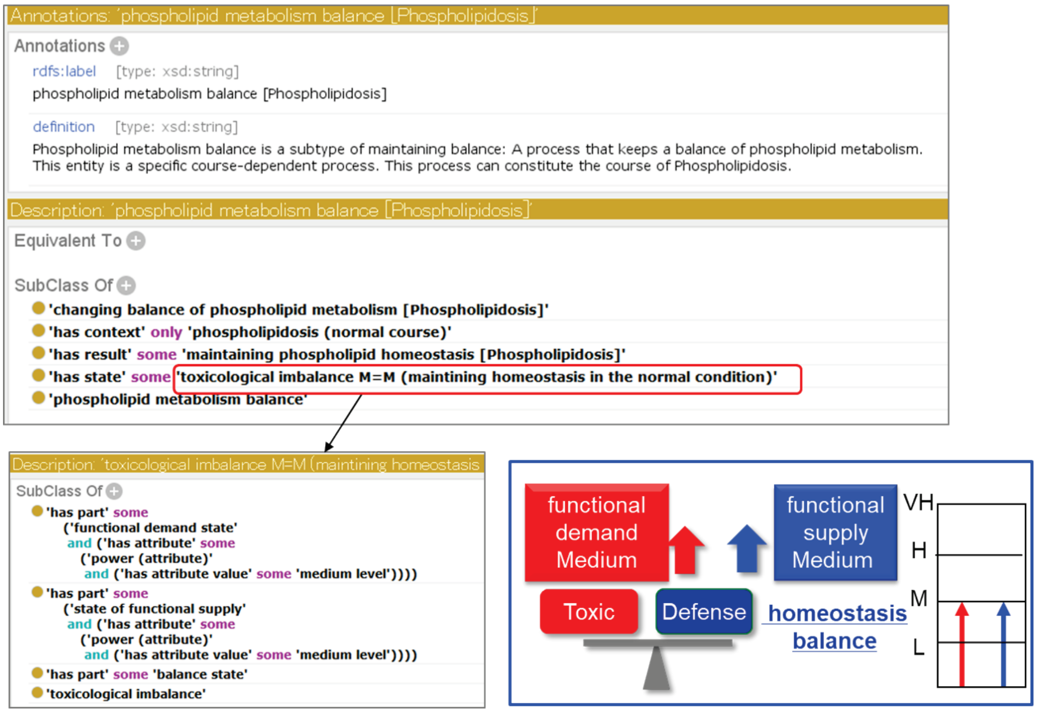


However, in the case of a phospholipid metabolic imbalance (moderate), the imbalance state is represented by a ‘toxic imbalance M <H.’
‘Toxic imbalance M <H’ has:
1) Medium level of the biological defense and 2) High level of functional demand as toxic action.

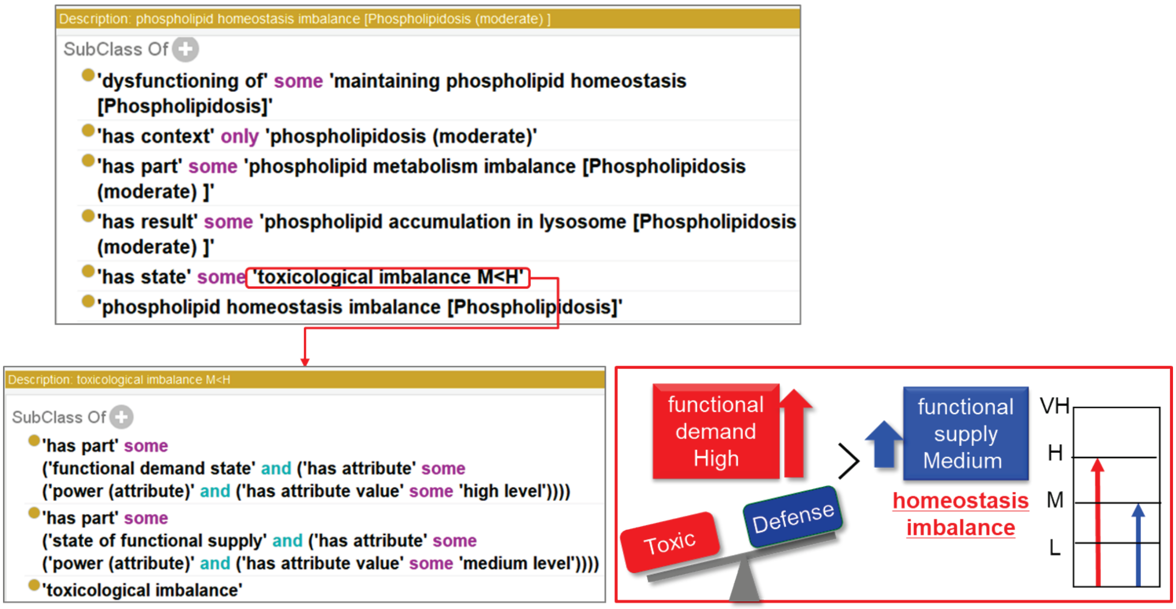

**Supplementary Information 3**

Supplementary Figure S1. TOXPIOT system architecture

- The RDF data are stored in an RDF triple store using Apache jena Fuseki to construct the SPARQL endpoint.
- Regarding the web application system for TOXPILOT, necessary information is dynamically acquired via SPARQL queries
- TOXPILOT generates graphs using D3.js of the JavaScript library

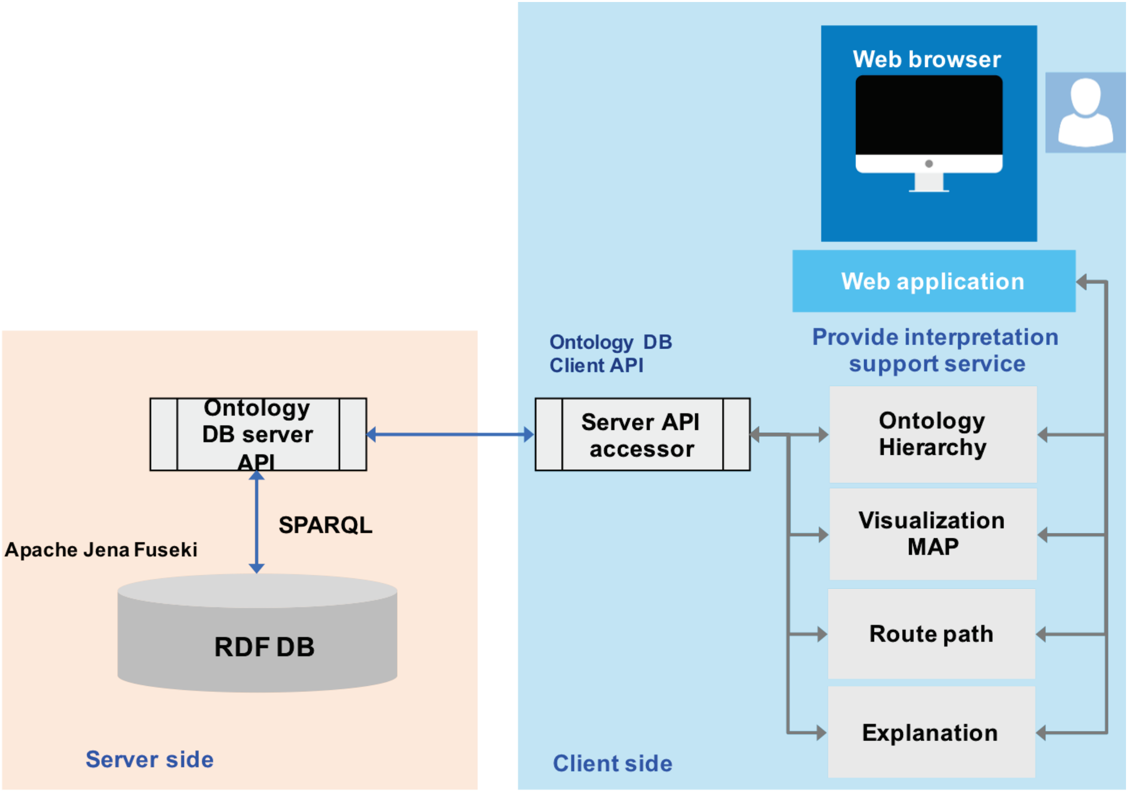

Supplementary Table S1 PubMed search list for hepatotoxic courses

| Toxic course | Search terms |
|---|---|
| ER stress | ("endoplasmic reticulum stress"[MeSH Terms] OR "unfolded protein response"[MeSH Terms]) AND "liver"[MeSH Terms] |
| Glutathione depletion | "glutathione"[MeSH Terms] AND "chemical and drug induced liver injury"[MeSH Terms] Sort by: PublicationDate |
| Ground glass degeneration | (ground[All Fields] AND ("glass"[All Fields])) AND "liver"[MeSH Terms] |
| Eosinophillic granular degeneration | ((peroxisomes[MeSH Terms] AND liver[MeSH Terms])) AND proliferation |
| Phospholipidosis | "phospholipidosis"[All Fields] AND "liver"[MeSH Terms] |
| Lipidosis | "fatty liver"[MeSH Terms] AND "chemical and drug induced liver injury"[MeSH Terms] |
| Hepatocarcinogenesis | "Carcinogenesis"[MeSH Terms] AND "liver"[MeSH Terms] |
| Hypertrophy | "hypertrophy"[MeSH Terms] AND "chemical and drug induced liver injury"[MeSH Terms] |
| Hepatic fibrosis | "fibrosis"[MeSH Terms] AND "chemical and drug induced liver injury"[MeSH Terms] |
| Necrosis | "necrosis"[MeSH Terms] AND "chemical and drug induced liver injury"[MeSH Terms] |
| Apoptosis | "apoptosis"[MeSH Terms] AND "chemical and drug induced liver injury"[MeSH Terms] Sort by: PublicationDate |
| Mitochondrial damage | "mitochondria, liver"[MeSH Terms] AND "chemical and drug induced liver injury"[MeSH Terms] |

Supplementary Schema S1. Examples of the TXPO development process

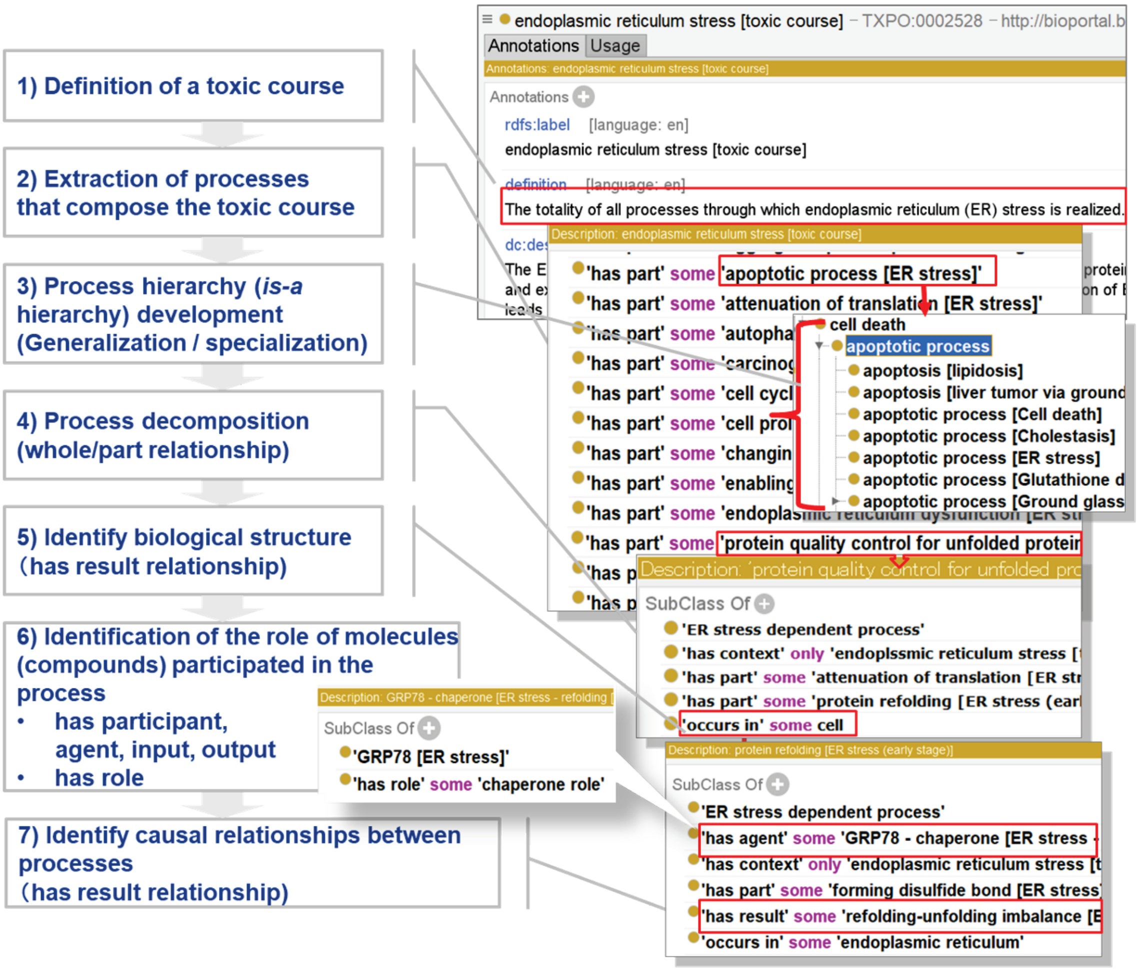

**Supplementary Information 4.** SPARQL query examples

Example1:

Find the cause(s) and result(s) of "increasing hepatocyte volume" in the course of "phospholipidosis (severe)."

```
PREFIX rdfs:<http://www.w3.org/2000/01/rdf-schema#>
PREFIX rdf:<http://www.w3.org/1999/02/22-rdf-syntax-ns#>
PREFIX owl:<http://www.w3.org/2002/07/owl#>
select distinct (<http://purl.obolibrary.org/obo/TXPO_0000921>
as ?focused) ?focused_label ?result ?result_label ?cause ?cause_label {
   <http://purl.obolibrary.org/obo/TXPO_0000921> rdfs:label ?focused_label.
{
   <http://purl.obolibrary.org/obo/TXPO_0000921> rdfs:subClassOf ?focused_blank.
   ?focused_blank owl:someValuesFrom ?result;
    owl:onProperty <http://purl.obolibrary.org/obo/TXPO_0000069>.
   ?result rdfs:label ?result_label.
 } union {
    ?cause rdfs:subClassOf ?cause_blank;
   rdfs:label ?cause_label.
   ?cause_blank owl:someValuesFrom <http://purl.obolibrary.org/obo/TXPO_0000921>;
   owl:onProperty <http://purl.obolibrary.org/obo/TXPO_0000069>.
 }
}
```

| URI | Description |
|---|---|
| http://purl.obolibrary.org/obo/TXPO_0000921 | increasing hepatocyte volume [phospholipidosis] |
| http://purl.obolibrary.org/obo/TXPO_0000069 | has result |

| Valiable | Description |
|---|---|
| ?focused | Focused process |
| ?focused_label | Label of focused process |
| ?focused_blank | Blank node for the focused process assigned to have 'has result' relation |
| ?result | Result process |
| ?result_label | Label of result process |
| ?cause | Causation process |
| ?cause_label | Label of causation process |
| ?cause_blank | Blank node for the cause assigned to have 'has result' relation. |

Example2:
Find the generalized process of “increasing hepatocyte volume in phospholipidosis”, sibling processes, and their dependent courses.

```
PREFIX rdfs:<http://www.w3.org/2000/01/rdf-schema#>
PREFIX rdf:<http://www.w3.org/1999/02/22-rdf-syntax-ns#>
PREFIX owl:<http://www.w3.org/2002/07/owl#>
select distinct ?parent ?parent_label ?sibling ?sibling_label ?course ?course_label {
 <http://purl.obolibrary.org/obo/TXPO_0000921> rdfs:subClassOf ?parent.
 ?sibling rdfs:subClassOf* ?parent.
  ?parent rdfs:subClassOf* <http://purl.obolibrary.org/obo/TXPO_0000145>;
   rdfs:label ?parent_label.
  ?sibling rdfs:label ?sibling_label.
 ?course rdfs:subClassOf+ <http://purl.obolibrary.org/obo/TXPO_0000009>;
  rdfs:label ?course_label;
  (rdfs:subClassOf/owl:someValuesFrom)+ ?sibling.
 FILTER(!isBlank(?parent))
 FILTER(!isBlank(?sibling))
}
```

| URI | Description |
|---|---|
| http://purl.obolibrary.org/obo/TXPO_0000921 | increasing hepatocyte volume [phospholipidosis] |
| http://purl.obolibrary.org/obo/TXPO_0000145 | function-related process |
| http://purl.obolibrary.org/obo/TXPO_0000009 | toxic course |

| Valiable | Description |
|---|---|
| ?parent | Parent of the focused process |
| ?parent_label | Label of the parent process |
| ?sibling | Sibling of the focused process |
| ?sibling_label | Label of the Sibling |
| ?course | Toxic course which has the sibling process |
| ?course_label | Label of the toxic course |

Example3:
Find the sibling processes of "increasing hepatocyte volume in phospholipidosis" and their cause(s) and the result(s).

```
PREFIX rdfs:<http://www.w3.org/2000/01/rdf-schema#>
PREFIX rdf:<http://www.w3.org/1999/02/22-rdf-syntax-ns#>
PREFIX owl:<http://www.w3.org/2002/07/owl#>
select distinct ?parent ?parent_label (<http://purl.obolibrary.org/obo/TXPO_0000921>
as ?focused) ?focused_label ?course ?course_label ?sibling ?sibling_label ?result ?result_label ?c
ause ?cause_label {
{
  <http://purl.obolibrary.org/obo/TXPO_0000921> rdfs:subClassOf ?parent;
   rdfs:subClassOf+ <http://purl.obolibrary.org/obo/TXPO_0001715>;
   rdfs:label ?focused_label.
  ?parent rdfs:subClassOf+ <http://purl.obolibrary.org/obo/TXPO_0000145>;
   rdfs:label ?parent_label.
  ?sibling rdfs:subClassOf* ?parent;
   rdfs:label ?sibling_label.
  ?course rdfs:subClassOf+ <http://purl.obolibrary.org/obo/TXPO_0000009>;
   rdfs:label ?course_label;
   (rdfs:subClassOf/owl:someValuesFrom)+ ?sibling.
 } optional {
  ?sibling rdfs:subClassOf ?sibling_blank.
  ?sibling_blank owl:someValuesFrom ?result;
   owl:onProperty <http://purl.obolibrary.org/obo/TXPO_0000069>.
  ?result rdfs:label ?result_label.
 } optional {
   ?cause rdfs:subClassOf ?cause_blank;
  rdfs:label ?cause_label.
  ?cause_blank owl:someValuesFrom ?sibling;
  owl:onProperty <http://purl.obolibrary.org/obo/TXPO_0000069>.
 }
}
```

| URI | Description |
|---|---|
| http://purl.obolibrary.org/obo/TXPO_0000921 | increasing hepatocyte volume [phospholipidosis] |
| http://purl.obolibrary.org/obo/TXPO_0001715 | toxic course dependent process |
| http://purl.obolibrary.org/obo/TXPO_0000145 | function-related process |
| http://purl.obolibrary.org/obo/TXPO_0000009 | toxic course |
| http://purl.obolibrary.org/obo/TXPO_0000069 | has result |

| Valiable | Description |
|---|---|
| ?parent | Parent of the focused process |
| ?parent_label | Label of the parent process |

| | |
|---|---|
| ?focused | Focused process |
| ?focused_label | Label of the focused process |
| ?course | Toxic course which has the focused or sibling process |
| ?course label | Label of the toxic course |
| ?sibling | Sibling of the focused process |
| ?sibling_label | Label of the sibling process |
| ?sibling_blank | Blank node for the sibling process assigned to have ‘has result’ relation |
| ?result | Result process |
| ?result_label | Label of result process |
| ?cause | Causal process |
| ?cause_label | Label of result process |
| ?cause_blank | Blank node for the cause assigned to have ‘has result’ relation |
